# Understanding and addressing mental health challenges of families admitted to the neonatal intensive care unit

**DOI:** 10.1038/s41372-024-02187-9

**Published:** 2024-12-07

**Authors:** Ashley D. Osborne, Daphna Yasova Barbeau, Tiffany Gladdis, Kara Hansen, Tonia Branche, Emily R. Miller, Christine C. Pazandak, Margaret K. Hoge, Michelle Spencer, Diana Montoya-Williams, Ryan Barbeau, Heather Padratzik, Stephen Lassen

**Affiliations:** 1 Division of Neonatal-Perinatal Medicine, Shawn Jenkins Children’s Hospital, Department of Pediatrics, Medical University of South Carolina, Charleston, SC, USA; 2 Envision Physician Services, Davie, FL, USA; 3 Department of Pediatrics, University of Missouri-Kansas City, Kansas City, MO, USA; 4 Children’s Mercy Hospital, Kansas City, MO, USA; 5 Department of Maternal-Fetal Medicine, University of Missouri-Kansas City, Kansas City, MO, USA; 6 Division of Neonatology, Ann and Robert H. Lurie Children’s Hospital; Department of Pediatrics, Northwestern University, Feinberg School of Medicine, Chicago, IL, USA; 7 Division of Neonatology, Cincinnati Children’s Hospital; Department of Pediatrics, University of Cincinnati, Cincinnati, OH, USA; 8 Division of Neonatal-Perinatal Medicine, Department of Pediatrics, UT Southwestern Medical Center, Dallas, TX, USA; 9 Division of Neonatology, University of Tennessee College of Medicine, Chattanooga, TN, USA; 10 Department of Pediatrics, University of Tennessee College of Medicine, Chattanooga, TN, USA; 11 Division of Neonatology, Children’s Hospital of Philadelphia; Department of Pediatrics, Perelman School of Medicine, University of Pennsylvania, Philadelphia, PA, USA; 12 Santa Fe College, Gainesville, FL, USA; 13 Parent of a Neonatal Intensive Care Unit Graduate, St. Louis, MO, USA; 14 Department of Pediatrics, University of Kansas Medical Center, Kansas City, KS, USA; 15 These authors contributed equally: Ashley D. Osborne, Daphna Yasova Barbeau.

## Abstract

This article reviews the psychological distress experienced by NICU families, including anxiety, postpartum depression (PPD), and post-traumatic stress disorder (PTSD), in addition to providing recommendations for clinicians at the individual, institutional, and national level. Currently, mental health screenings, specialized evaluations, and treatment options are not routinely offered to NICU families and are frequently under-utilized when offered. Here we provide expert opinion recommendations to address challenges in supporting universal screening, offering bedside interventions, including trained mental health professionals in care plans, updating neonatology training competencies, and advocating for policies that support the mental health of NICU families. We advocate that mental health of NICU families be incorporated into the standard of care.

## INTRODUCTION

An admission to the NICU is a traumatic event. Parents of infants admitted to the NICU describe increased mood, anxiety, and post-traumatic stress (PTS) symptoms compared to parents without a NICU admission [1]. These complex emotions impact parents during and after admission and have implications for parent-child bonding, lactation, family dynamics, and perceptions of the child’s health for years after discharge [1–4]. In this article, we briefly examine the known effects of emotional strain on NICU parents. We propose best practice recommendations based upon expert opinion for universal NICU parent mental health (MH) screening and targeted interventions. These recommendations are based upon current knowledge and represent a crucial first step to incorporating parental MH support within the standards of care for neonatology.

## OVERVIEW OF MENTAL HEALTH CONCERNS IN THE NICU

### Mental health of NICU mothers

Perinatal mood and anxiety disorders (PMADs), including diagnoses of anxiety, depression, or other mood disorders that occur during pregnancy and/or through one year postpartum, are some of the most common conditions identified in pregnancy, and rates of PMADs are increasing [5, 6]. NICU mothers have increased anxious, depressive, and PTS symptoms compared to mothers whose infants are not in the NICU [1, 3, 5, 7, 8]. Forty to fifty percent of NICU mothers experience postpartum depression (PPD) compared to approximately 10–15% of mothers of term healthy infants worldwide [9–11]. Earlier gestational age, low birthweight, lack of social support, and ongoing infant illness and disability are risk factors for prolonged depressive symptoms [10]. Additionally, meta-analyses demonstrate that greater than 40%-85% of NICU mothers experience anxiety or PTS symptoms in the first postpartum month [1, 3]. Fortunately, rates of anxiety and PTS appear to decrease over time; however, symptoms persist in about 25% of mothers at one year after birth [1, 3].

### Mental health of NICU fathers

Fathers of NICU infants have greater stress and anxiety than fathers of non-NICU infants and are at increased risk for depression and PTSD [12–14]. Greater than 60% of NICU fathers scored above the threshold for depression in the first week of their infant’s life [12]. In the NICU, fathers may be left out of medical updates, counseling, and education resulting in them feeling excluded from caregiving and decision-making [15–17]. Frequently, they are tasked with managing the home, caring for other children, and continuing to work during the NICU admission. This may contribute to isolation and a perceived lack of control [13, 14, 18].

### Mental health considerations for high-risk populations

Many factors can predict an individual to be at higher risk for emotional distress. These factors include: prior MH problems and trauma, low social support, young age, less education, substance use/abuse, non-white race, poverty, recent immigration, and multiple births in addition to “objective measures of poor infant health” such as extremely low birth weight and prolonged admission [10, 19, 20]. To further compound the issue, low-income and minoritized women are less likely to receive care for PPD, less likely to receive follow-up care, and less likely to fill prescriptions related to MH [19, 21, 22]. Careful attention is warranted to the MH of Black caregivers as many describe experiencing racism at multiple levels during the NICU admission [23].

PMADs are not only seen in parents of the most premature infants; parents of late preterm and term infants in the NICU also suffer from increased depression and anxiety, and frequently remain underrepresented in studies, screening, and interventions aimed at NICU parents [7, 19, 24, 25]. Meta-analyses indicate length of stay plays a marginal role in parental MH [6]. Severity of infant illness also does not fully predict parental stress [6, 8]. Admission to the NICU itself, with disruption of the anticipated parent role, is the significant stressor [6].

There remains a paucity of data regarding MH outcomes of fathers, co-mothers, extended family, and stepparents in the NICU; although the available data indicate partners do experience increased MH symptomatology [26, 27]. Furthermore, there has been little research about the experiences of lesbian, gay, bisexual, transgender and gender nonconforming individuals in the NICU. These populations continue to be affected by barriers to care such as poor provider knowledge, stigma, and inadequate access, despite known disparities in MH and obstetric care [28–30]. Notably, insights to support LGBTQ-headed families in the NICU have been proposed [31]. Parents with limited English proficiency have also been understudied, though it is well understood that language barriers contribute to suboptimal healthcare delivery, including MH services [32]. Lastly, adoptive parents may have specialized needs that are unrecognized in the NICU [33].

### Long-term effects of poor parental mental health

The lasting impact of parental MH on child development and parent-child relationships is well-established. Unaddressed MH symptoms in NICU parents have long term consequences. Parents may continue to experience symptoms past the perinatal period and tragically, maternal suicide accounts for up to 20% of postpartum deaths [34]. Stress can cause less secure parent-infant attachment and higher scores of PTS symptoms which are correlated with bonding failure [35–37]. The infant is also at increased risk for their own health, developmental, psychological, and behavioral concerns [38]. For example, parent stress and anxiety is linked to lower child cognitive function in the first few years of life, especially if accompanied by low social support [39]. Maternal depression is associated with child maladjustment, delayed growth, and lower IQ [40]. Even subclinical level of psychiatric symptoms can interrupt bonding and impair childhood development [41]. Finally, untreated parental MH symptoms can lead to development of the Vulnerable Child Syndrome, where caregivers perceive previously high-risk children as more susceptible to illness and injury than they truly are [42]. High perceived vulnerability scores are associated with increased emergency room visits, separation difficulty, school underachievement, sleep problems, adolescent MH disorders, and behavioral problems [42–44].

Parents who experienced a NICU admission may experience relationship strain years after discharge with increased rates of divorce, which is further increased in the setting of children with disability [40]. Depression in one spouse increases the risk of depression in the other partner. In addition, siblings are at higher risk for depression [4, 45, 46]. Poor MH has broad effects on families; it is linked to lower income and higher unemployment in NICU parents, as well as fewer years of maternal education [45].

Finally, a rarely discussed consequence of stress in the peripartum period is impaired lactogenesis. This is particularly important in the care of critically ill neonates and requires close attention and intervention [47, 48]. These consequences likely only represent a fraction of the adverse effects of NICU admissions on families. For these reasons, NICU professionals should consider support of the entire family as vital to the optimal care of the infant.

## SCREENING FOR PSYCHOLOGICAL DISTRESS

There is no nationally accepted standard for MH screening or access to support in United States NICUs [49, 50]. The American Academy of Pediatrics (AAP) encourages PPD screening at 1-, 2-, 4- and 6-month routine pediatric visits [51]; however, many NICU infants remain hospitalized at these times, resulting in missed screening opportunities. A study found that under 50% of NICUs are routinely providing screening for PPD or MH symptoms [52]. Universal screening is necessary as psychological distress is frequently unanticipated and undetected [53, 54]. By relying on clinical suspicion of staff or self-report by a parent, the majority of those in need are missed [55].

### How & when to screen

While there is ongoing debate regarding screening tool, timing, and inclusion criteria, screening parents at risk for PMADs improves likelihood of treatment, especially when a mental health professional (MHP) is embedded in the unit [49, 56]. Newer screening tools are simple, quick, and effective and have been studied in NICU mothers [19, 49, 52, 54]. Some reliable screening tools are only 2–4 questions in length and can easily be integrated into routine care [5, 19, 49, 56]. The Patient Health Questionaire-2 (PHQ-2) is a depression questionnaire that has two questions and has been effectively used in the NICU and postpartum settings [55, 57]. The Psychosocial Assessment Tool– NICU (PAT-NICU) is a new screening measure and the first to evaluate multiple domains of psychosocial risk (social support, stress reactions, family resources) [58]. Table 1 includes brief descriptions of selected screening tools [57–68]. Individual NICUs should select the most appropriate tool for their population. Special considerations should be given to the time needed to complete a screening tool and languages in which the tools are validated.

Notably, recommendations for screening in the NICU for emotional distress have been previously proposed [49]. These include screening primary caregivers at least twice during admission. The first screen should occur “early” by 1–2 weeks after admission to allow families time to adjust to the shock and logistics of a NICU admission [49], and another screen should be performed within 48 h of discharge. Studies have demonstrated that parents may experience significant symptoms of distress as early as two weeks postpartum [11], while anecdotal reports indicate parents may experience an increase in symptoms near discharge. By screening at these timepoints, staff will be more likely to identify caregivers who would benefit from additional support.

Unfortunately, most NICUs lack dedicated psychosocial staff [69]. Some NICUs have found success utilizing nurses to administer screenings, while others have benefited from the involvement of lactation consultants and case managers [54]. Utilizing electronic HIPPA-compliant modalities to screen may help overcome barriers of staff availability. There is precedent for successful documentation of a maternal PMAD screen within the child’s electronic health record [70].

### Screening follow-through

Screening alone is insufficient to meet parental MH needs. Optimally, MHPs would be integrated into every NICU team, building rapport with families across transitions of care, screening parents and providing timely and personalized interventions. Unfortunately, this optimal integration is not always available; although, it should be the standard of care. In the interim, it is imperative that units establish a plan to follow-through on all screens. Parents should receive results from all screenings regardless of results (positive or negative). When a parent screens positive, a secondary screen should be conducted by a MHP within a couple days. If suicidal ideation or concern for their child’s safety is reported, a same day referral to a MHP with expertise in evaluation and management of MH emergencies must be ensured. This is an especially vulnerable time and mismanagement of these symptoms can cause additional harm. If a MHP is not available within the hospital system, a robust referral system to community resources needs to be in place. Table 2 lists a selection of MH resources. A number of states are also creating referral networks to ensure appropriate access to a perinatal MHPs such as PeriPAN [71] in Texas. Another resource that might be available for non-emergent needs is a counselor or MHP within the practice that gave obstetric care to the mother during pregnancy. An additional strategy is to partner with social work, psychology, or marriage and family therapy training programs.

### Creating a successful screening program

Implementing a screening program can be challenging; however, a few key factors for success have been identified. The components of an effective screening program should delineate which screening tool to use, who administers the screen and follows-up results, when to screen, and how to use the results [72]. Successful programs have program champions, multidisciplinary staff support and engagement, and incorporate screening into existing workflows [54]. Program champions, especially with protected time devoted to screening implementation, can ensure continuity, and decrease the burden on other staff.

Other barriers include lack of leadership support and MH resources. Programs with inadequate leadership support demonstrate lower screening rates [73]. However, the most significant barrier to implementing an effective screening program is lack of MH services [54]. NICU staff may find success by partnering with community resources or a hospital-based clinical psychologist to enhance referrals and provide immediate psychosocial support [49, 54]. Many cities have federally qualified health centers with behavioral health services and psychology departments that provide psychotherapy on a sliding fee scale.

## INTERVENTIONS TO ADDRESS PARENTAL MENTAL HEALTH

### Organizational practices

Studies show that simple interventions, like staff-parent interactions, can buffer feelings of stress and trauma [74, 75]. Information-sharing increases parental empowerment and participation in the care of their infants. The converse is also true; negative interactions with staff increase feelings of anxiety and decrease engagement in care [74, 75]. What is said to parents, and how it is said, impacts well-being. Recommendations and online training aimed toward improving sensitive, compassionate communication have been published and should be included in staff onboarding and neonatology training programs [76–78]. Notably, a course for neonatal fellows on providing psychosocial support to NICU families has been proposed [79].

NICUs may have varied experiences promoting parental MH due to inherit differences such as staffing models, funding, and access to community resources. Regardless, all NICUs can engage in approaches that support families by practicing family-centered care (FCC) and TIC. FCC, a multifaceted healthcare delivery philosophy that respects the family as an integral part of the healthcare team, includes: day-to-day practices (parent education, shared decision-making, parental care involvement), NICU design, support groups, technology-based support (live-stream video cameras, SMS messaging) and discharge preparation [80]. TIC is a care model that recognizes past trauma affects families, their NICU experience, and infant outcomes. Six TIC principles to promote parent attunement have been described: safety, trustworthiness and transparency, peer support, collaboration, empowerment, and awareness of cultural, historical and gender issues [81]. Various FCC and TIC initiatives have demonstrated a positive impact on parental MH and are promising interventions [80–83].

### Peer support

Peer support groups positively affect parental coping and parent-infant interactions [84]. A designated support person can help parents feel more informed, less stressed, and better prepared for discharge [83, 85]. A parent “buddy” program found that mothers assigned a peer buddy reported less stress at 4 weeks and less anxiety and depression at 16 weeks compared to controls [83]. Matching peer support based on an infant’s medical condition, ethnicity/language, and location may be necessary for this benefit [83, 86]. Importantly, recruited peer volunteers need to have effectively integrated their NICU experience, and should receive screening and training to prevent any potential harm to themselves or their peers [85].

### Bedside intervention

There are several activities that parents can participate in at the bedside to promote their own mental wellbeing including “skin-to-skin” care (SSC) and infant massage. SSC has several benefits which include improving parent competence, increasing infant attachment, decreasing levels of maternal stress and PMAD symptoms, and decreasing paternal fears of harming their fragile infant [80, 87]. Infant massage has also been shown to have benefits on maternal MH and maternal-child relationships [88]. In addition, interventions that focus on mindfulness have been shown to decrease stress, anxiety, and depression [89, 90].

### Educational-behavioral programs and therapies

Educational-behavioral programs such as the Creating Opportunities for Parent Empowerment (COPE) and the Mother-Infant Transaction Program (MITP) were found to significantly reduce psychological distress in parents of preterm infants [82, 91, 92]. Meta-analyses have found evidence that Cognitive Behavioral Therapy (CBT) decreases maternal depressive symptoms and empowerment programs positively affect parental MH [57, 93, 94]. Brief, focused programs on prevention of traumatic stress have also demonstrated success in improving maternal emotional distress [92, 95]. CBT interventions aimed at anxiety, depression, and traumatic stress in NICU parents have all been successful in reducing MH symptoms [92]. Pilot data from an ongoing randomized controlled trial aimed at decreasing parental perceptions of child vulnerability, anxiety and depression found that manualized CBT is feasible, can be delivered with high fidelity, and deemed helpful by parents [96]. While these interventions are effective when given to one individual parent, there is also promise for group therapy sessions and telehealth interventions, but further research is needed.

## ADVOCACY INITIATIVES & FUTURE DIRECTIONS

For interventions to have a positive long-standing impact, we need to extend our care beyond the bedside. Table 3 addresses advocacy recommendations at an individual, institutional, and national level to ameliorate MH symptoms. Due to competing responsibilities, it is challenging for clinicians to adequately support parental MH. In an ideal scenario, there would be at least one full-time licensed clinical social worker and licensed psychologist per 20 beds [49]. Notably, guidelines for essential skills of psychologists working in NICU settings have been proposed and a growing network of NICU psychologists exists [97, 98]. More recent designation surveys of NICUs ask if there is a parental MH screening program. Parental MH screening is a part of pediatric residency and fellowship training guidelines, which suggests the need for more systematic NICU parental MH efforts for both designation and training purposes.

Effective strategies to leverage more MH resources at the level of hospital administration begins with documenting the need locally by performing screening. Secondly, it is imperative to underscore the value provided by MHPs to justify the additional cost of this staff [82, 99, 100]. Additional research in the NICU specifically may still be needed to persuade local administrators, and creating a gold standard may be valuable for hospitals to align on this initiative.

There may be a discrepancy in connecting with mothers based on whether they have public versus private insurance. Mothers with public insurance compared to those with private insurance may visit the NICU less frequently and thus be screened at lower rates for MH distress due to challenges with transportation, childcare, finances, and fewer maternity leave benefits [76]. Additionally, parents may not qualify for treatment at certain centers due to their outpatient status or may have trouble finding a provider who accepts their insurance or who practices in their area; even those with insurance may have difficulty securing coverage for MH concerns [101]. There is a large paucity of trained MHPs in most settings. Training to become a licensed MHP is long and rigorous, requiring years to complete the necessary graduate degrees, supervised experiences, and licensure exams. These issues highlight the importance of advocating for local and national policy changes that expand postpartum benefits, improve reimbursement, and expand MH services. These policies are overdue and necessary for NICUs to follow current recommendations to better support families.

A significant opportunity to support families is through improved parental leave policies. Extended and paid maternity leave practices in Europe yielded significant MH benefits that extended beyond the perinatal period for decades [102]. Studies indicate having less than 8-12 weeks of leave are associated with increased depressive symptoms [103]. Further, the World Health Organization released 2022 recommendations that “parental leave and entitlements address the special needs of mothers, fathers and other primary caregivers of preterm or low-birth-weight infants” [104]. Lack of childcare may be a barrier to caregivers’ physical presence in the NICU and contribute to the lack of following through with referrals [76]. Access to affordable childcare could decrease this significant and financial stressor, especially for families of low socioeconomic status.

There is growing evidence that a variety of interventions enhance parental emotional wellbeing in the NICU. Telehealth or technology-driven MH assessments and treatments are on the horizon, but research is needed to examine these methods. More studies investigating cost-effective programs, different models of peer support, best practices for implementation of universal screening programs (i.e., screening timing, modalities, methods), and how to best support caregivers other than the birth parent are needed.

## CONCLUSION

Stress and MH disorders are common in parents with infants admitted to the NICU. This predisposes them to long-term MH concerns in addition to poorer physical health. Furthermore, parental MH impacts bonding, attachment, lactogenesis, parenting style, and long-term neurodevelopment of their infants. Unfortunately, routine screening, specialized evaluation, and treatment options are not routinely offered in the NICU, even though well-validated screening tools exist to identify at-risk parents and many interventions show promise at decreasing symptoms. Therefore, it is imperative that units develop a screening program linked with appropriate resources and referrals, and that neonatal professionals advocate for the MH needs of NICU families and family-friendly public policies.

## Figures and Tables

**Table 1.** Selected mental health screening tools.

Selected mental health screening tools				
	Target population	Description	Language(s)	Sensitivity/Specificity
**Depression Measures**				
Edinburgh Postnatal Depression Scale (EPDS)(54)	Postpartum mothers	• 10-item self-report • Free	Multiple	0.85/0.84
Postpartum Depression Screening Scale (PDSS)(55)	Postpartum mothers	• 35-item self-report • Requires training to administer • $35-150	English, Spanish, Italian	0.91/0.72
Beck Depression Inventory (BDI)(56)	Mothers & fathers	• 21-item self-report • $100-150	English, Spanish	0.81/0.92
Patient Health Questionnaire-9 (PHQ-9) (57)	Mothers & fathers	• 9-item self-report • Free	English, Spanish	0.88/0.88
Patient Health Questionnaire-2 (PHQ-2) (100)	Mothers & fathers	• 2-item self-report • Free	English, Spanish	0.82/0.87
**Anxiety Measures**				
General Anxiety Disorder-7 (GAD-7)(58)	Mothers & fathers	• 7-item self-report • Free	Multiple	0.92/0.76
Beck Anxiety Inventory (BAI)(59)	Mothers & fathers	• 21-item self-report • $100-150	Multiple	0.85/0.88
Edinburgh Postnatal Depression Scale-Anxiety (EPDS-A)(60)	Postpartum mothers	• 3-item subscale of EPDS • Free	Multiple	0.67/0.88
**Trauma Measures**				
Perinatal PTSD Questionnaire-II (PPQ-II)(61)	Mothers	• 14-item self-report • Free	Multiple	Sensitivity 0.82
City Birth Trauma Scale (City BiTS)(62)	Mothers & fathers	• 29-item self-report • Free	Multiple	Cronbach alpha = 0.92
Impact of Events Scale- Revised (IES-R) (63)	Mothers & fathers	• 22-item self-report • Free	Multiple	Cronbach alpha = 0.84
Posttraumatic Stress Disorder Checklist (PCL-5)(64)	Mothers & fathers	• 20-item self-report • Free	Multiple	0.74/0.70
**Family Psychosocial Risk**				
Psychosocial Assessment Tool (PAT-NICU)	Mother & fathers	• 13-item self-report • Free	English	0.43/0.70

**Table 2.** Electronic and phone mental health resources for NICU parents.

Electronic & phone resources for NICU parents		
Organization	Website/Phone Number	Description
988 Suicide and Crisis Hotline	Phone: 988	The “911” for mental health emergencies
Hand to Hold	www.handtohold.org	Support groups, peer support and 1:1 counseling
Mammha	www.mammha.com	Offers digital screening and emergency referrals
March of Dimes	www.marchofdimes.org www.shareyourstory.org	Peer community support, parent & provider resources
Massachusetts General Hospital’s Center for Women’s Mental Health	https://womensmentalhealth.org	Outpatient & inpatient treatment by state, parent & provider resources
Mobile apps	Betterhelp: www.betterhelp.com Moodfit: www.getmoodfit.com Sanvello: www.sanvello.com Talkspace: try.talkspace.com UCLA Mindful: https://www.uclahealth.org/marc/ucla-mindful-app	Telehealth therapy Customizable tools to improve mood Peer support, coaching, telehealth therapy Telehealth therapy Guided meditations
National Maternal Mental Health Hotline	833-9-HELP4MOMS (43-5746)	Free counselors via phone or text (English & Spanish with access to interpreters for 60 languages)
National Perinatal Association	www.nationalperinatal.org/mental-health	“Mental health plan” materials, hotline numbers
National Suicide Prevention Lifeline	800-273-TALK (8255)	Free crisis support
Postpartum Progress	https://postpartumprogress.com	Peer community support
Postpartum Support International	www.postpartum.net Phone: 800-944-4773 Text: 503-894-9453	Toll-free helpline, online support groups (English & Spanish), parent & provider resources

Curated alphabetical list of national resources for parental peripartum mental health support, not an exhaustive list.

**Table 3.** Expert recommendations to support parents’ mental health in the NICU.

**Recommendations for Individual Clinicians**
Be familiar with the mental health risks associated with NICU admissions and understand these risks persist beyond the neonatal period
Understand that parental mental health impacts long-term infant development and outcomes
Recognize that non-birthing parents/caregivers have underrecognized mental health needs and they should be treated as equal caregivers to birthing parents
Acknowledge that high-risk populations for mental health conditions need further levels of support
Normalize the range of caregiver reactions to having an infant admitted to the NICU
Be aware of the evidence-based practices that can mitigate parental distress and facilitate parent involvement at the bedside
Be familiar with resources to enhance family support in your community
Understand how the NICU admission is disruptive to the entire family unit and strive to incorporate practices that support the entire family and their unique cultural needs
Assess families for psychosocial needs and refer any concerns to appropriate mental health professionals
Include families in decision making for your unit, your community, and research design
**Recommendations for Institutions**
Establish a universal screening program for parental psychological distress
Foster connections with existing community organizations, create opportunities for peer (parent-to-parent) support, and consider implementation of emotional-behavioral programs and therapies (e.g. COPE, CBT interventions) to promote parental mental health
Employ mental health professional(s) in the NICU
Incorporate mental health awareness, knowledge, and communication skills into neonatology training programs
**Recommendations on a National Level**
Develop standardized policies for parental mental health screening and treatment
Advocate for extended, universal, paid parental leave, accessible and affordable childcare, and comprehensive access to health care
Attention and funding to provide equity in mental health resource utilization
Support scholarly work surrounding screening and treating psychosocial distress in NICU parents and use of technology-assisted assessments/therapy
